# Mining SNPs in extracellular vesicular transcriptome of *Trypanosoma cruzi*: a step closer to early diagnosis of neglected Chagas disease

**DOI:** 10.7717/peerj.2693

**Published:** 2016-11-24

**Authors:** Pallavi Gaur, Anoop Chaturvedi

**Affiliations:** 1 Center of Bioinformatics, Institute of Inter Disciplinary Studies, Nehru Science Center, University of Allahabad, Allahabad, Uttar Pradesh, India; 2 Department of Statistics, Nehru Science Center, University of Allahabad, Allahabad, Uttar Pradesh, India

**Keywords:** Extracellular vesicles, Chagas, RNA-Seq, NGS data analysis, R/Bioconductor, Biomarkers, SNPs, *T. cruzi*, Neglected disease, Bioinformatics

## Abstract

One of the newest and strongest members of intercellular communicators, the Extracellular vesicles (EVs) and their enclosed RNAs; Extracellular RNAs (exRNAs) have been acknowledged as putative biomarkers and therapeutic targets for various diseases. Although a very deep insight has not been possible into the physiology of these vesicles, they are believed to be involved in cell-to-cell communication and host-pathogen interactions. EVs might be significantly helpful in discovering biomarkers for possible target identification as well as prognostics, diagnostics and developing vaccines. In recent studies, highly bioactive EVs have drawn attention of parasitologists for being able to communicate between different cells and having likeliness of reflecting both source and target environments. Next-generation sequencing (NGS) has eased the way to have a deeper insight into these vesicles and their roles in various diseases. This article arises from bioinformatics-based analysis and predictive data mining of transcriptomic (RNA-Seq) data of EVs, derived from different life stages of *Trypanosoma cruzi*; a causing agent of neglected Chagas disease. Variants (Single Nucleotide Polymorphisms (SNPs)) were mined from Extracellular vesicular transcriptomic data and functionally analyzed using different bioinformatics based approaches. Functional analysis showed the association of these variants with various important factors like Trans-Sialidase (TS), Alpha Tubulin, P-Type H+-ATPase, etc. which, in turn, are associated with disease in different ways. Some of the ‘candidate SNPs’ were found to be stage-specific, which strengthens the probability of finding stage-specific biomarkers. These results may lead to a better understanding of Chagas disease, and improved knowledge may provide further development of the biomarkers for prognosis, diagnosis and drug development for treating Chagas disease.

## Introduction

Having the potential of playing important roles in various diseases, extracellular vesicles (EVs) are one of the most primarily focused areas in today’s biology. Although introduced in late 80’s (Johnstone et al., 1987) as wasted vesicles and later as a significant role player in other complex functions like antigen presentation (Raposo et al., 1996), EVs are now confirmed as the mediators in intercellular exchange of genetic material in eukaryotes and multicellular organisms (Belting & Wittrup, 2008). By composition, they are mainly composed of lipid rafts (Théry et al., 1999; Wubbolts et al., 2003), membrane trafficking proteins (Hemler, 2003), transmembrane molecules, T cell-stimulating molecules, signaling molecules, and also small RNAs; mRNA and miRNA (Théry, Zitvogel & Amigorena, 2002; Valadi et al., 2007). These micros to nano sized vesicles have ability to circulate in intercellular milieu through almost every body fluid (Raposo & Stoorvogel, 2013).

EVs can be categorized according to the path/mechanism they choose to be released in the extracellular milieu. They can either be exosomes, which are 30–100 nm cup-shaped vesicles, released by exocytic fusion of multivesicular bodies (MVBs) and plasma membrane or ectosomes which are larger vesicles ranging around 100–1,000 nm in diameter and released directly from the plasma membrane by budding. Additionally, a third category may belong to apoptotic bodies, which are even larger than aforementioned categories having diameter of more than 1 μm and found to be originating from apoptotic cells (György et al., 2011).

Functionally, EVs can either be immune-stimulatory or immune-suppressive, depending on the cells they originate from. In previous studies, when it became clear that EVs were involved in transporting small RNAs (Valadi et al., 2007) that might be translated into proteins by recipient cells (Ratajczak et al., 2006; Valadi et al., 2007), the research regarding EVs exploded immensely. Other studies show that the contents of EVs, like extracellular RNAs (ExRNAs) are highly stable (Valadi et al., 2007; Skog et al., 2008) and represent both target and source environment (Choi et al., 2012; Sandvig & Llorente, 2012). Also, extracellular miRNAs (EXmiRNAs) are found to be capable of modulating gene expression in recipient cells (Mittelbrunn et al., 2011). Upon fusion of EVs with a target cell, alteration in the biology of the target tissue, through heterogeneous bioactive cargo is observed in many studies (Mittelbrunn et al., 2011; Montecalvo et al., 2012; Buck et al., 2014; Twu et al., 2013). Since proteins and RNAs carried by EVs are protected from hydrolysis or degradation in extracellular environment, they are considered to be a potential source of disease biomarkers (Keller et al., 2011).

Later, it was observed that parasites also release EVs (Silverman et al., 2010; Marcilla et al., 2012; Twu et al., 2013; Bernal et al., 2014); thus, hypothesis of involvement of these vesicles in host-pathogen interaction and immune response grew stronger. In fact, EVs extracted from *Heligmosomoides polygyrus*, a gastrointestinal nematode, have recently been shown to alter gene expression in host cells and suppress innate immune responses in mice (Buck et al., 2014).

These significant properties of EVs make them promising in therapeutic field of neglected diseases, Chagas disease included. Chagas disease, also called American Trypanosomiasis, is caused by the parasite *T. cruzi*. The triatomine bug (kissing bug), a bloodsucking insect that feeds on humans and animals, spreads this parasite through its feces. *T. cruzi* has distinct life stages which consist of three main developmental forms. The non-infective epimastigotes are found in the midgut of the bug, where they multiply by binary fission. Epimastigotes move to the hindgut and differentiate into metacyclic trypomastigotes that have capacity to infect mammalian cells. When the parasite enters the body, the trypomastigotes circulate in the blood, but do not divide. The trypomastigotes move to the cytoplasm and transform into amastigotes. The amastigotes, after many rounds of division, again transform back into trypomastigotes and enter the bloodstream, where they may invade cells in mammalian body or be transmitted to the insects during their meal of blood.

In a previous study, it`s been shown that *T. cruzi* releases at least two types of EVs; ectosomes and exosomes, generated by distinct pathways (Goncalves et al., 1991). In a study carried out in 2013, it was found that infective metacyclic forms release vesicles that carry virulence factors such as GP82 glycoproteins and mucins, while in contact with HeLa cells (Bayer-Santos et al., 2013). This suggests the possibility that EVs can be used as nano-carriers to deliver virulence and modulatory factors into the host cells. Furthermore, EVs have full potential to be used as delivery system for drugs, proteins, miRNAs/siRNAs, and other molecules (Fais et al., 2013). Jang & Gho (2014) showed that if EVs can be bio-engineered, then there is a great hope of target delivering of therapeutic agents which can be immensely helpful in revolutionizing vaccine development for Chagas disease. Since transcriptomic data enriches the information regarding small RNAs (miRNAs) which are key players in gene regulation (Ghildiyal & Zamore, 2009), studies regarding EVs transcriptomic data would help exploring different aspects of Chagas disease.

In this regard, another study was carried out in 2014 to analyze EVs extracted from *T. cruzi* (Bayer-Santos et al., 2014). In this study, EVs were extracted from epimastigotes and metacyclic trypomastigotes forms (clone Dm28c; two biological replicates) of *T. cruzi*. Total RNA extracted from both replicates was mixed before small RNA isolation, to obtain sufficient amount of small RNA. This procedure was performed for all sample-replicates. Briefly, the small RNA fraction of 16–40 nt was isolated from total RNA of metacyclic trypomastigote parental cells (mCell), epimastigote (eVes) and metacyclic-derived vesicles (mVes). Illumina GAIIx was used for sequencing cDNA library which was purified from Illumina TrueseqTM small RNA preparation kit.

This research article elaborates the analysis and predictive data mining of high throughput transcriptomic data produced by aforementioned RNA-Seq study carried out in 2014. The analysis included important steps of filtration of raw reads, mapping reads to transcriptome and detection of variants. Further, these variants were classified according to their types (Single Nucleotide Polymorphisms (SNPs), Multiple Nucleotide Polymorphisms (MNPs), indels and complex events) and functionally analyzed. It was found that the transcripts consisting of these variants encode various important proteins that have vital role in the pathogenesis, prognosis and diagnosis of Chagas disease. Some of the putative candidate variants were found to be present only in specific stage of *T. cruzi* that strengthens the hope of finding stage specific biomarkers. Since EVs have a pivotal role in host-pathogen interaction, this study would help researchers to have a better understanding of the roles and significance of EVs in Chagas disease.

## Materials and Methods

### Data and pre-processing

Data were downloaded from Sequence reads Archive (SRA), NCBI in fastq format having accession no. SRX433186, SRX433187 and SRX433188. All the three files contained raw reads of three bio-samples, extracted from EVs belonging to different life stages of *T. cruzi.* Firstly, data were subjected to quality check which was done by scripting on R/Bioconductor using ‘ShortRead’ (Morgan et al., 2009) package. Suitable filters were applied for refining the data. In parallel, quality check was also done using a popular tool, FastQC (Andrews, 2010). Fastx (http://hannonlab.cshl.edu/fastx_toolkit/) which is command line based tool, was used to remove the adapter and filter the low quality reads. Information about the adapter was retrieved by contacting author of source work. We set Phred score to 30 as minimum qualifying score for reads and performed subsequent analysis with high quality reads only. Phred score given by −10log10 p + 64 represents base quality where p is the confidence of the base calling program (Ewing et al., 1998). In pre-processing, raw reads passed through various filtering steps like checking per base quality, filtering duplicate sequences, discarding Ns (no base assigned during base call) etc.

### Mapping reads to genome/transcriptome

Transcriptomic reads require specialized algorithm for mapping that can justify reads arisen from exon-exon junction. TopHat2 (Trapnell, Pachter & Salzberg, 2009) was used for mapping high quality reads from all the samples (eVes, mVes and mCell) against reference (REF) transcriptome downloaded from database TriTrypDB 4.0 (http://tritrypdb.org/tritrypdb) (Aslett et al., 2010). TopHat2 is a fast splice junction mapping package for RNA-Seq reads that maps non-junction reads (those contained within exons) in first step using Bowtie (http://bowtie-bio.sourceforge.net) (Langmead et al., 2009) and then indexes the REF genome/transcriptome using a technique called Burrows–Wheeler transform (Burrow & Wheeler, 1994). The output of mapping; Sequence Alignment Map (SAM) file was viewed using viewer ‘Tablet’ (Milne et al., 2010), whereas SAMtools (Li et al., 2009) commands were executed for checking the statistics of mapping and conversion of SAM format into its binary format called Binary Alignment Map (BAM).

### Detection of variants

For identifying variants in all samples, Freebayes; (Garrison & Marth, 2012) a haplotype based variant detector was used. This command line based tool used alignment (BAM) file and the REF transcriptome as input and eventually delivered the results in a specific ‘Variant Call Format (VCF).’ VCF is basically composed of two parts; Header and variant call record. The header contained information about the dataset, REF genome and different statistical factors with their annotations while variant call record consisted of details of variants along with all scores/information secured by each statistical/biological factor.

### Filtering the VCF output

The filtration of variants was important in order to avoid false positive variants. The credibility of variants depends on many determinants, details of which are delivered by VCF file in forms of various fields like ‘quality of variants (QUAL),’ ‘read depth (DP),’ ‘sum of quality of alternate allele’ (QA) etc. Some fields in VCF file as ‘identity’ (ID), ‘genotype’ (GT), ‘reference’ (REF), ‘alternate’ (ALT), ‘contig,’ ‘genomic coordinates,’ etc., deliver biological particulars of variants. To make more confident variant calls in mapped data, we filtered out the low-quality variants that failed the criteria of DP > 10 and QUAL > 30. Since, QUAL is phred scaled probability of variation (REF/ALT) indicating its existence at that particular site, the criterion of QUAL > 30 was expected to be a safe criterion. Additionally, a sharp inspection of QA was also done while filtering the variant files. Since reads having a phred score below 30 were already discarded in pre-processing step, these aforementioned criteria produced high quality results for subsequent analysis.

### Mining putative candidate variants and their functional annotations

The filtered variants from all the samples were checked for their GTs which informed about the alleles carried by the sample at that very site. It is encoded by 0 for the REF allele, whereas 1 for first ALT allele. If there is a single ALT allele, GT can be either of following types; (1) 0/0: Homozygous REF. (2) 0/1: Heterozygous i.e. one copy of each REF and ALT alleles. (3) 1/1: Homozygous ALT. After this, with the help of TriTrypDB 4.0 database (http://tritrypdb.org/tritrypdb) putative SNPs were tagged and checked if they were coding/non-coding, synonymous/non-synonymous along with their strand positions and encoded genes. We also studied functional aspects of these variants and performed Gene Ontology (GO) on encoded genes for enrichment analysis.

## Results and Discussion

### A careful pre-processing results in a significant mapping

Raw data having width of 56 cycles contained 6729968, 10382407 and 5475574 reads in eVes, mVes and mCell, respectively. Qualities of all the samples were rechecked after pre-processing and found to be better. Mapping of all the samples with seed length 15 and only one allowed mismatch resulted in 69.6% mapping of eVes, 74.8% mapping of mVes and 57.3% mapping of mCell.

### Putative candidate variants rely on various criteria

**Figure 1:**
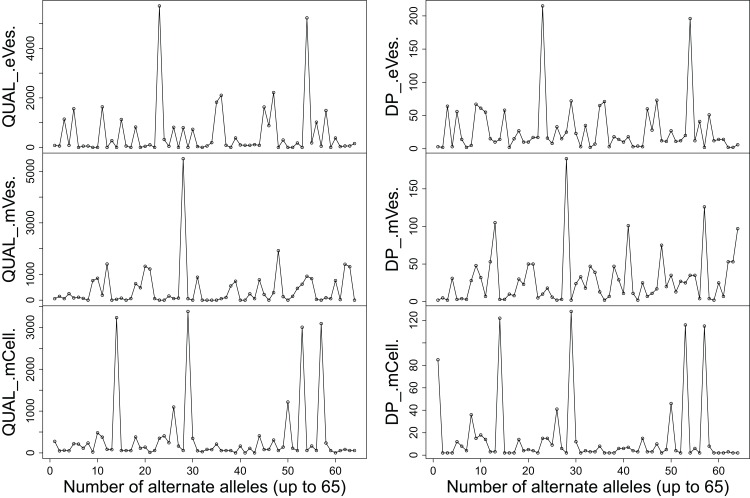
Plots showing (A) QUAL and (B) DP of all samples.

**Figure 2:**
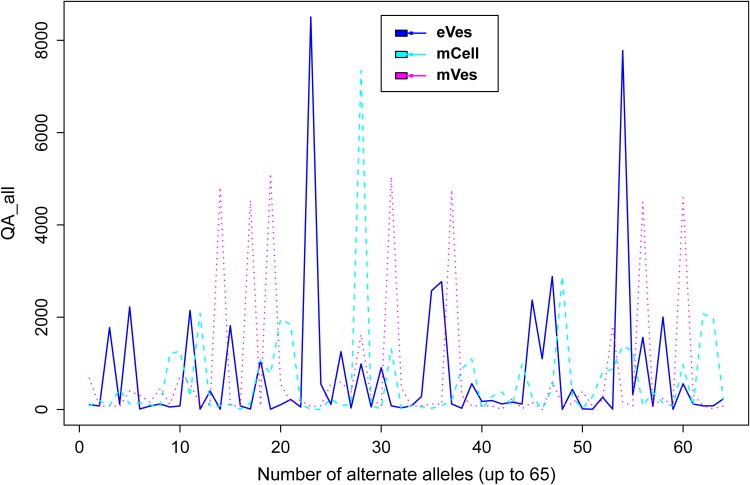
Plot showing QA of all samples.

Filtration of the VCF files with aforementioned criteria resulted in putative candidate variants. A consistent pattern was observed between QUAL and DP for every sample (Fig. 1). Since, QA is also an important criterion to make a confident variant call, a plot elaborating QA of all variants was also drawn (Fig. 2) by scripting on language R.

We filtered VCF files not only on the basis of one but combinations of many criteria, thus increasing the credibility of the resultant variants. Collectively, these criteria like DP, phred quality and quality of ALT allele etc also helped in detecting the false positive variants. Finally, from eVes derived ExRNAs, total 66 variants were found to be putative while 128 and 67 variants were believed to be confident variants from infective mVes and mCell derived samples, respectively. Since, variants might include many types of variations including SNPs, MNPs, insertions, deletions and complex variants; we further observed these types in all the samples. SNPs were found to be the most common type of variant among all (Table 1). This is worth mentioning here that *T. cruzi* is considered to be a diploid organism, so information on GTs of various variants was a crucial factor to take into account. With respect to REF allele, one ALT allele was found to be present with each variant which is in accordance with nature of a diploid organism. These ALT alleles were found to be present in considerable frequency with a considerable number of reads supporting them. Mostly, variants were found to be homozygous for the ALT allele but the highest percentage of heterozygous variants i.e. variants with one copy of each REF and ALT alleles was found to be associated with vesicles derived from eVes and mVes (Table 2).

**Table 1:** Number of different types of variants found in each sample.

Samples	SNPs	MNPs	Deletions	Insertions	Complex events	Total variants
eVes	50	5	3	5	3	66
mVes	96	9	5	10	8	128
mCell	58	4	0	2	3	67

**Table 2:** Percentage of different types of variant genotypes found in each sample.

Samples	Heterozygous variants	Homozygous ALT	Homozygous REF
eVes	11 (∼17%)	38 (∼57%)	17 (∼26%)
mVes	22 (∼17%)	83 (∼65%)	23 (∼18%)
mCell	6 (∼9%)	57 (∼85%)	4 (∼6%)

### Classification of candidate variants enriched the significance of non-coding RNAs

Using genome annotations, we found that putative variants belonged to small RNA sequences derived from a variety of non-coding RNAs such as rRNA, tRNA, snoRNAs along with exons (coding-sequences) and pseudogenes (Table 3). The most abundant class was rRNA whereas snoRNA class was abundant in parent cell and metacyclic developmental form. It has been shown in a previous study that rRNA-derived small RNAs are functional molecules processed by special enzymes (Li et al., 2012). This study also showed the significance of other non-coding RNAs. Specifically, there have been evidences that *T. cruzi* produces tRNA-derived small RNAs (tsRNAs) (Garcia-Silva et al., 2010; Franzén et al., 2011; Reifur et al., 2012) that may get delivered to susceptible mammalian cells (Garcia-Silva et al., 2014). In a study, it was shown that synthetic *T. cruzi* tsRNAs were able to modify gene expression on transfection of host HeLa cells (Garcia-Silva et al., 2014). Thus, variants found in tRNAs of infective state may affect the regulatory pathways to a significant extent.

**Table 3:** Percentage of variants belonging to different classes of small RNAs.

Samples	rRNA (%)	snoRNA (%)	tRNA (%)	CDS (%)	Pseudogenes (%)
mCell	∼41	∼24	∼11	∼8	∼16
eVes	∼71	∼4	∼0	∼16	∼9
mVes	36	24	4	30	6

### Functional annotation of putative candidate variants

Further characterization of variants led us to elucidate their effects on various gene products. We investigated whether variants were coding/non-coding and synonymous/non-synonymous. With the help of TriTrypDB 4.0 database, the variants were also analyzed for their encoded genes and products. We found some significant gene products, associated with candidate variants (Table 4). Here, we succinctly discuss how these gene products play significant roles in Chagas disease.

**Table 4:** Affected gene products from putative candidate variants.

Samples	Coding variants	Non-coding variants
eVes	Alpha Tubulin putative gene IV, Trans-Sialidase (TS) group and hypothetical protein	Ribosomal RNA large subunit alpha
mVes	TS group, P-type H+-ATPase, Retrotransposon Hot Spot (RHS) pseudogene and hypothetical protein	C/D snoRNA, ribosomal RNA large subunit (beta 3′ and 5′ partial), ribosomal RNA small subunit (3′ partial)
mCell	TS group V and VI, P-type H+-ATPase, RHS	C/D snoRNA family, H/ACA snoRNA

‘Alpha Tubulin,’ associated with SNP (SNP ID–NGS_SNP.Tcruzi_11788.1297, Gene ID–TcCLB.411235.9) in our results, has already been found to be eliminated in the urine of infected host (Bertot et al., 1998). Although, being synonymous, presence of this SNP in non-infective stage may raise the possibility of prognosis through EVs because synonymous mutation can also alter the structure, function and expression level of protein along with cellular response to therapeutic targets.

‘Trans-Sialidase’ (TS) is believed to be a potential therapeutic target as well as an important role player in the pathogenesis in Chagas disease (Mendonça-Previato et al., 2010; Miller & Roitberg, 2013). TS is required by the triatomine bug (Nagamune et al., 2004) and found to be associated with SNPs belonging to different life stages of *T. cruzi*.

Having a role in acidification of the endocytic pathway of *T. cruzi* (Vieira et al., 2005), ‘P-Type H+-ATPase’ was found to be associated with variants of infective stage (mVes) derived vesicles. H+-ATPases maintain low pH of the cell microenvironment and are essential for the fusion of EVs with target cells. This implies that EVs can be used as a target to affect the H+-ATPase driven proton pump to diminish the acidic microenvironment that in turn, can lead the diminishment of infective cells. If we examine the composition of EVs released from infected host fluids, we can examine the presence of H+-ATPase that can be targeted as drug responder.

The sequenced telomeric regions from *T. cruzi* contain the enriched presence of Retrotransposon Hot Spot (RHS) genes in subtelomeric regions along with the presence of ‘TS’ genes at telomeres. This suggests that *T. cruzi* chromosomal ends could have been the site of generation of new gp85 variants which is an important adhesion molecule in invasion of mammalian cells by *T. cruzi* (Kim et al., 2005). EVs from mVes and mCell both contained signatures of hypothetical proteins in conserved form which implies that EVs might be useful in drug development if extracted from both the bug as well as human host. An earlier research, focusing on this problem screened an epimastigote-subtracted trypomastigote cDNA expression library by genetic immunization in order to find new vaccine candidates for Chagas disease. This approach led to the identification of a pool of 28 putative gene fragments and their sequence analysis revealed that 19 out of 28 genes were hypothetical proteins or un-annotated *T. cruzi* open reading frames, which certainly would not have been identified by other methods of vaccine discovery (Tekiel et al., 2009). Here, these hypothetical proteins were found to be associated with transcriptome of EVs thus providing a great path for further development of prognosis and vaccine for Chagas disease.

Variants in non-coding regions also were found to be associated with important factor like rRNA modulation. ‘C/D’ and ‘H/ACA’ are known to be involved with methylation and pseudouridylation of mammalian rRNA nucleotides, respectively. Having these snoRNAs in EVs probably confirms that EVs are involved in the processing of many RNA substrates. Since EVs are involved in cell to cell communication, it can be interesting to extract EVs from blood of the infected host and investigate for the mRNAs and miRNAs to find out the genes associated with these aforementioned kinds of enzymes/proteins that can be used as biomarkers. Since the molecular components of EVs play important roles in intercellular signaling and may be involved in disease progression, the profiling of EVs will not only lead to further understand the molecular mechanism but also aid in the discovery of novel diagnostic biomarkers. Also, it would be fruitful to explore and analyze full network of the small RNAs and protein expression behavior in EVs from both the vector and host, and comparing them with those of normal.

Besides the annotated SNPs, freebayes reported some other novel variants also which qualified the filter criteria successfully. For example, candidate SNP with Gene ID–TcCLB.403903.40 (genomic position–43) obtained from mCell stage strongly indicates a heterozygous SNP. Likewise, there are many other variants that strongly show the probability of being present at that particular genomic position range. All the details of these variants can be found (describing all the criteria) in supplementary data along with details of other results generated at various steps with graphical representations (see the figshare dataset).

### GO enrichment analysis of putative candidate variants shows their involvement in mRNA turnover

GO enrichment analysis was performed on all genes encoded by variants. They belonged to almost similar cellular component—‘vesicles’ which validated the source origin of raw data. Genes associated with SNPs of non-infective stage showed ontology with molecular functions like GTP binding, GTPase activity and structural molecule activity whereas genes associated with SNPs of infective stage showed ATPase activity coupled to transmembrane transporter activities. Literatures show that GTPases are important in membrane trafficking and they might get involved in impairment or hindrance of EVs secretion (Hsu et al., 2010). Furthermore, GTP binding has been associated with the mRNA turnover (Woo et al., 2011). These raise the assumption that EVs may regulate the mRNA turnover and modulate the cell environment in non-infective stage too.

It is worth mentioning here that other than SNPs, microsatellites also have capabilities to elucidate the genetic architecture of complex traits. Since, previous studies have shown the usefulness of microsatellite analysis in *T. Cruzi* with respect to population genetics and phylogenetic analysis (Oliveira et al., 1998; Macedo et al., 2001; Martínez et al., 2013), it would be interesting to analyze microsatellite genotyping in EVs’ transcriptome of *T. Cruzi*. Also, it would be fruitful to explore microsatellites in other kinetoplastids’ EVs to have a better overview of phylogenetic makeup. Although, whole microsatellite analysis falls out of this article’s scope, it could be noticeable that in our primary search for microsatellites in EVs’ transcriptome of infective and non-infective stage of *T. Cruzi*, we found some dinucleotide repeats having minimum 6 bp length with at least 20 bp of flanking regions. STR-FM (Fungtammasan et al., 2015) on Galaxy web resource (http://usegalaxy.org/) (Afgan et al., 2016) was used to conduct this detection. We found dinucleotide repeats having length of no more than 7 bp but the repeats were mostly same as reported by previous studies (Oliveira et al., 1998; Martínez et al., 2013), details of which can be found in the figshare dataset.

## Conclusion

Exploring the EVs from *T. cruzi* suggests that the RNA containing vesicles may be determinant for various biological processes, including cell to cell communication and pathogenesis. This bioinformatics based analysis gives a functional insight into the putative roles of candidate variants present in EVs of *T. cruzi*, where some of the SNPs were stage specific that gives a hope to find stage specific biomarkers. Putative SNPs resulted from this study may be further experimentally checked for having influence on gene expression, transcript stability and phenotype that may contribute in initiation or progression of Chagas disease. These SNPs may also be used to check the responses of certain drugs. Chagas disease, being a neglected disease, demands an unprecedented introspection into possible prognosis, diagnosis and drug development. The information achieved by this study would help researchers to propel their focus in field of EVs, not only for Chagas disease but also for other neglected diseases.

Furthermore, the experimental validation of candidate variants that included both; the annotated and novel variants would provide a solid foundation for association genetics studies. The functional annotation, classification and gene ontology enrichment highlighted various properties of the candidate variants that would help in further studies; for example coding SNPs found in stage specific EVs can be used for further gene variation analysis. This gene variation analysis would explore the amino acid substitutions that perhaps lead to functional differences, which later can be associated with phenotypic effects. The analysis also showed the signatures of conserved hypothetical proteins in EVs transcriptome of *T. cruzi* that provide a path for further investigation of vaccine candidate. This bioinformatics based analysis suggests that full profiling of small RNAs, genetic variants and protein expression behavior of EVs extracted from infected host would be very fruitful in multiple ways. Because of the potential importance of EVs, identification of SNP-based markers would rapidly benefit large-scale analysis of Chagas disease. Since, EVs from body fluids carry RNAs that offers access to the transcriptome of the host organism, the comparative as well as association study of EV transcriptome of the vector and host of Chagas disease would lead to a better understanding of the underlying molecular mechanism of Chagas disease.

## Additional Information and Declarations

### Data Deposition

The following information was supplied regarding data availability: Gaur P. 2016. Figshare. https://dx.doi.org/10.6084/m9.figshare.3838038.v1 Raw data available on SRA, NCBI with accession no. SRX433186, SRX433187, SRX433188.
